# Avaliação Eletrocardiográfica de Recém-Nascidos Normais na Primeira Semana de Vida – Estudo Observacional

**DOI:** 10.36660/abc.20210843

**Published:** 2022-08-24

**Authors:** Marina de Souza Pimenta, Nelson Samesima, Carlos Alberto Pastore, Vera Lucia Jornada Krebs, Gabriela Nunes Leal, Werther Brunow de Carvalho

**Affiliations:** 1 Hospital das Clínicas Faculdade de Medicina Universidade de São Paulo São Paulo SP Brasil Instituto da Criança (ICr) – Hospital das Clínicas da Faculdade de Medicina , Universidade de São Paulo , São Paulo , SP – Brasil; 2 Hospital das Clínicas Faculdade de Medicina Universidade de São Paulo São Paulo SP Brasil Instituto do Coração (InCor) – Hospital das Clínicas da Faculdade de Medicina , Universidade de São Paulo , São Paulo , SP – Brasil

**Keywords:** Eletrocardiografia, Miócitos Cardíacos, Recém-Nascido

## Abstract

**Fundamento:**

O período neonatal é marcado por muitas alterações importantes no sistema cardiovascular, principalmente na primeira semana de vida. Diferentemente da população adulta, estudos sobre dados de eletrocardiograma (ECG) no período neonatal são escassos. Este é o primeiro estudo a descrever alterações eletrocardiográficas em uma coorte de recém-nascidos com ecocardiogramas normais.

**Objetivos:**

Analisar padrões eletrocardiográficos de uma população de recém-nascidos a termo, sem anomalias morfológicas ou funcionais cardíacas, e comparar os resultados com a literatura.

**Métodos:**

Neste estudo observacional, ecocardiogramas e resultados de ECG de 94 neonatos divididos em três grupos etários (até 24 horas, entre 25 e 72 horas, e entre 73 e 168 horas de vida) foram avaliados e comparados com aqueles descritos por Davignon et al. Um valor de p < 0,05 foi considerado estatisticamente significativo.

**Resultados:**

Diferenças significativas na direção da onda T foram detectadas nas derivações V1 (p= 0,04), V2 (p= 0,02), V3 (p= 0,008) e V4 (p= 0,005). Houve diferenças entre nossos resultados e a literatura atual na maioria dos parâmetros.

**Conclusão:**

Recém-nascidos a termo com menos de 24 horas de vida apresentaram significativamente mais ondas T positivas que aqueles com mais horas de vida. Encontramos muitas diferenças nos parâmetros de ECG em comparação aos descritos por Davignon et al., particularmente nas amplitudes de P, Q, R, S, duração do QRS, R/S e R+S. Esses achados indicam a necessidade de mais estudos para uma interpretação definitiva do ECG em recém-nascidos.

## Introdução

O período neonatal é marcado por muitas alterações anatômicas e hemodinâmicas, especialmente na primeira semana de vida, quando ocorre a transição do padrão de circulação fetal para neonatal. ^1 , 2^ No feto, a placenta é um leito vascular de baixa resistência, e o ventrículo direito (VD) é o ventrículo dominante, responsável por aproximadamente 60% do débito cardíaco. O coração trabalha com uma carga de trabalho quase constante, com circulação de alto volume e baixa resistência. Após a secção do cordão umbilical e a primeira respiração, ocorrem diminuição da resistência vascular pulmonar e aumento na resistência vascular sistêmica. A pressão e o fluxo no VD diminuem e a pós-carga aumenta quando a placenta é removida, e o débito do ventrículo esquerdo (VE) aumenta em duas vezes com o aumento no fluxo de sangue pulmonar. O *foramen ovale* e o *ductus arteriosus* se fecham, e a predominância ventricular muda do VD para o VE, com subsequente aumento no tamanho e número dos cardiomiócitos. ^1 , 3^

Não se sabe se essas alterações circulatórias nos primeiros dias de vida podem levar a diferentes padrões no eletrocardiograma (ECG). Alterações hemodinâmicas são avaliadas na prática clínica por meio de parâmetros clínicos (frequência cardíaca, saturação de oxigênio, padrão respiratório, auscultação cardíaca) e exames complementares (p.ex. ecocardiograma, lactato sérico, e bicarbonato de sódio). Contudo, diferentemente da população adulta, estudos eletrocardiográficos no período neonatal são escassos. ^4^

O objetivo deste estudo foi descrever os achados eletrocardiográficos em neonatos a termo sem malformações cardíacas e função cardiovascular normal durante a internação e compará-los com os achados descritos por Davignon et al. ^2^ Este é o primeiro estudo correlacionando os achados eletrocardiográficos com ecocardiograma normal em uma coorte de neonatos.

## Métodos

### População

Neste estudo observacional, entre agosto de 2016 e julho de 2018, foram avaliados resultados de ECG e ecocardiogramas de neonatos em seus primeiros sete dias de vida (168 horas), todos nascidos em uma unidade neonatal terciária em São Paulo, Brasil.

### Aspectos éticos

O estudo foi aprovado pelo Comitê de Ética do Hospital das Clínicas da Faculdade de Medicina da Universidade de São Paulo (aprovação número CPE 272/13/2016; CAPPesq 1.662.356) e conduzido de acordo com a Declaração de Helsinki. O comitê de ética dispensou o estudo da necessidade de um termo de consentimento, uma vez que os exames de ECG e ecocardiograma são rotinas na unidade neonatal.

Os critérios de inclusão foram idade gestacional (IG) entre 37 e 41 semanas e seis dias, e menos de 169 horas de idade pós-natal. Malformações cardíacas foram excluídas por ecocardiograma nas primeiras 169 horas de vida. Todos os recém-nascidos apresentaram função cardiovascular normal durante a hospitalização.

Recém-nascidos com malformações não cardíacas importantes, tais como anormalidades neurológicas e cromossômicas, IG inferior a 37 semanas ou maior ou igual a 42 semanas, ou com anormalidades no ecocardiograma tais como malformações cardíacas (cardiopatias congênitas, disfunção valvar, defeito do septo, coarctação da aorta), hipertensão pulmonar persistente, limitações funcionais, e evolução cardiovascular anormal durante hospitalização foram excluídas.

Os recém-nascidos forma divididos por idade pós-natal em três grupos: até 24 horas, entre 25 e 72 horas, e entre 73 e 168 horas de vida para possibilitar a comparação de nossos achados com os descritos por Davignon et al. ^2^

### ECG de 12 derivações

**Figura 1:**
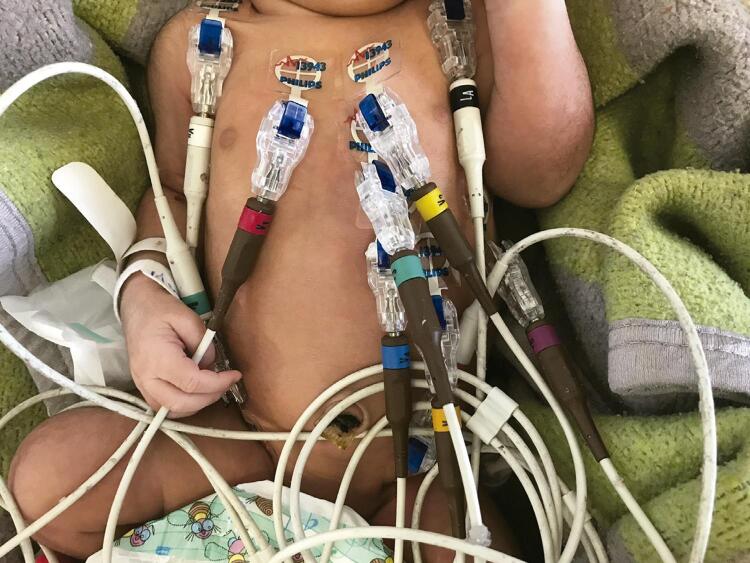
Posicionamento dos eletrodos do eletrocardiograma em recém-nascidos.

ECG de 12 derivações (Philips PageWriter TC20©, Koninklijke Philips, N.V.) foi realizada e analisada em todos os recém-nascidos por um único investigador, treinado, não cego. Eletrodos com gel sólido adesivo foram posicionados sobre os ombros direito e esquerdo, cristas ilíacas e V1-V6 como recomendado por diretrizes ^5^ ( Figura 1 ). Os ombros e as cristas ilíacas foram escolhidos em vez dos braços e pernas devido à movimentação corporal intensa natural do recém-nascido, a fim de se reduzir ruídos e melhorar sinais do ECG. ^6^

Os seguintes parâmetros foram avaliados:

- frequência cardíaca (bpm, automaticamente medida pelo aparelho),
- eixo QRS no plano frontal (°),
- amplitude (mm) e duração (ms) da onda P e duração do intervalo PR no DII (ms),
- amplitude de: onda Q em DIII, aVF e V5-V6, onda R em aVR, V1-V2 e V4-V6, e ondas S em V1-V2 e V4-V6 (mm),
- razão R/S em V1 e V6,
- duração do QRS, intervalos QT e QTc (corrigido pela fórmula de Bazzet) em V2 (ms), e
- duração da onda T (ms) e orientação (+ / -) em todas as 12 derivações.

### Ecocardiograma

Um ecocardiograma bidimensional com Doppler detalhado foi realizado em todos os indivíduos por um cardiologista pediátrico experiente de plantão. O equipamento usado foi um Philips CX50 (Koninklijke Philips N.V.), com transdutores multifrequenciais S8-3 e S12-4. Medidas ecocardiográficas em modo M do átrio esquerdo, VD, VE, parede posterior, e diâmetros diastólico e sistólico do VE foram obtidas seguindo-se as diretrizes da *American Society of Echocardiography* . ^7^ A fração de ejeção do VE foi obtida pelo método Teichholz e considerado normal se maior ou igual a 55%.

### Análise estatística

Características qualitativas das mães e recém-nascidos foram descritas em frequências relativas e absolutas. Características quantitativas foram descritas utilizando-se medidas de resumo (média e desvio padrão) para todos os indivíduos. ^8 , 9^ Parâmetros eletrocardiográficos foram descritos de acordo com doenças maternas tais como hipertensão (ausente, primária, gestacional, primária + gestacional), e diabetes mellitus (ausente, tipo 1, tipo 2, gestacional). Com base no peso ao nascer, os neonatos foram classificados como pequeno para a idade gestacional (PIG), adequado para a idade gestacional (AIG), e grande para a idade gestacional (GIG); os valores foram descritos como medidas de resumo, e comparados quanto às categorias de interesse por análise de variância ( *one-way* ANOVA), seguido por comparações múltiplas de Bonferroni quando p<0,05. ^10^

Foi realizado o teste de normalidade da distribuição de dados Kolmogorov-Smirnov, e a presunção de normalidade foi aceita para a maioria dos parâmetros avaliados. Como essa é uma presunção relativamente fraca da ANOVA, ela foi conduzida para todas as variáveis sem que houvesse perda de poder nas análises, uma vez que o teorema central do limite garante a normalidade da distribuição da média, mesmo sem normalidade dos dados.

Os parâmetros foram descritos em curvas de percentis e comparados com valores normais de acordo com os percentis descritos por Davignon et al. ^2^ As análises foram realizadas usando o programa IBM-SPSS para Windows versão 22.0. Um valor de p<0,05 foi considerado estatisticamente significativo.

## Resultados

Durante o período do estudo, houve 2883 nascidos vivos na unidade neonatal, 1916 nascidos a termo. Exames de ecocardiogramas foram conduzidos em 753 recém-nascidos; 310 eram nascidos a termo e, desses, 191 não apresentavam nenhuma alteração anatômica significativa.

O exame de ECG foi realizado em 113 recém-nascidos, e 19 foram excluídos por apresentarem importantes malformações não cardíacas, principalmente anomalias do sistema nervoso central ou síndromes genéticas. A série final do presente estudo foi composta 94 pacientes.

As características clínicas dos recém-nascidos estão apresentadas na Tabela 1 . Os percentis dos parâmetros eletrocardiográficos estudos estão descritos na Tabela 2 . Na comparação entre grupos por idade ( Tabela 3 ), os recém-nascidos com menos de 24 horas de vida tiveram uma proporção significativamente maior de ondas T positivas em comparação a recém-nascidos com mais horas de vida (25-72 horas e 73-168 horas) nas derivações V1 (p = 0,04), V2 (p = 0,02), V3 (p = 0,008), e V4 (p = 0,005).

**Tabela 1:** Características clínicas do recém-nascidos avaliados (n=94)

Variáveis	Número (%)
**Grupo etário (horas de vida)**	
≤24 horas	11 (12)
25-72 horas	46 (49)
73-168 horas	37 (39)
**Classificação (peso)**	
PIG	9 (10)
AIG	77 (82)
GIG	8 (8)
**Parto**	
Vaginal	31 (33)
Fórceps	9 (10)
Cesárea	54 (57)
**Sexo**	
Feminino	53 (56)
Masculino	40 (43)
Indeterminado	1 (1)
	Média (DP)
IG (semanas)	38,6 (1,1)
**Peso ao nascer (gramas)**	3184 (551)

IG: idade gestacional; PIG: pequeno para a idade gestacional; AGA: adequado para a idade gestacional; GIG: grande para a idade gestacional; DP: desvio padrão.

**Tabela 2:** Percentis dos parâmetros eletrocardiográficos

Parâmetro	<24horas de vida			24-72 horas de vida			73-168 horas de vida		

	5%	50%	95%	5%	50%	95%	5%	50%	95%
Frequência cardíaca (bpm)	92,14	122,09	152,04	98,91	122,72	146,53	102,18	131,05	159,92
Ampl P DII (mm)	0,04	0,11	0,18	0,06	0,13	0,21	0,06	0,13	0,21
PR DII (ms)	70,55	92,73	114,91	71,43	99,13	126,83	73,40	98,38	123,36
QT V2 (ms)	227,59	301,82	376,04	206,84	293,48	380,12	202,20	274,05	345,90
QRS axis ( ° )	54,61	126,36	198,12	61,59	128,75	195,91	58,09	134,44	210,79
Ampl Q DIII (mm)	0,03	0,41	0,79	0,05	0,34	0,64	0,02	0,36	0,70
Ampl Q aVF (mm)	0,00	0,30	0,65	0,00	0,23	0,47	0,00	0,27	0,59
Ampl Q V5 (mm)	0,00	0,10	0,28	0,00	0,04	0,15	0,00	0,10	0,29
Ampl Q V6 (mm)	0,00	0,13	0,32	0,00	0,06	0,18	0,00	0,12	0,31
Ampl R aVR (mm)	0,00	0,33	0,83	0,00	0,33	0,71	0,00	0,25	0,66
Ampl R V1 (mm)	0,15	1,25	2,35	0,42	1,13	1,85	0,38	1,13	1,88
Ampl R V2 (mm)	0,56	1,35	2,14	0,46	1,19	1,92	0,43	1,27	2,11
Ampl R V4 (mm)	0,76	1,74	2,71	0,76	1,57	2,38	0,78	1,57	2,37
Ampl R V5 (mm)	0,35	1,41	2,48	0,51	1,30	2,09	0,49	1,29	2,09
Ampl R V6 (mm)	0,39	1,26	2,12	0,36	1,17	1,98	0,43	1,14	1,85
Ampl S V1 (mm)	0,24	1,00	1,75	0,00	0,97	2,10	0,00	0,67	1,37
Ampl S V2 (mm)	0,57	1,40	2,23	0,15	1,34	2,52	0,12	1,08	2,04
Ampl S V4 (mm)	0,00	1,21	3,23	0,07	0,97	1,87	0,08	0,88	1,67
Ampl S V5 (mm)	0,00	0,77	2,10	0,00	0,75	1,59	0,09	0,62	1,15
Ampl S V6 (mm)	0,00	0,59	1,66	0,00	0,67	1,51	0,08	0,52	0,96
R/S V1	0,24	1,37	2,50	0,00	2,10	5,87	0,00	2,38	5,46
R/S V5	0,00	5,26	18,79	0,00	3,03	8,63	0,00	3,41	11,42
R/S V6	0,00	5,31	17,78	0,00	3,32	9,46	0,00	3,17	8,20
R + S V2 (mm)	-9,16	-0,46	8,25	-11,73	-1,44	8,86	-7,37	1,88	11,13
R + S V4 (mm)	-14,88	5,23	25,33	-3,11	5,98	15,07	-3,29	6,97	17,24
S V2 + R V5 (mm)	1,68	2,81	3,94	1,19	2,64	4,09	1,14	2,38	3,61
S V1 + R V6 (mm)	1,01	2,25	3,50	0,72	2,14	3,55	0,88	1,81	2,73
Dur QRS V5 (ms)	40,00	40,00	40,00	30,60	44,78	58,97	27,41	46,49	65,56

Ampl: amplitude; bpm: batimentos por minuto; Dur: duração; mm: milímetros; ms: milissegundos.

**Tabela 3:** Parâmetros eletrocardiográficos de onda T por grupo etário

Variável	Horas de vida				p

	≤24	25-72	73-168		
Onda T em V1. n (%)					0,04
Positiva	5 (45)	8 (17)		2 (5)	
Negativa	3 (27)	23 (50)		23 (62)	
Minus-plus	3 (27)	15 (33)		12 (32)	
Onda T em V2. n (%)					0,02
Positiva	6 (54)	7 (15)		4 (11)	
Negativa	4 (36)	21 (46)		23 (62)	
Minus-plus	1 (9)	18 (39)		10 (27)	
**Onda T em V3. n (%)**					0,008
Positiva	8 (73)	12 (26)		5 (13)	
Negativa	2 (18)	15 (33)		20 (54)	
Plus-minus	0 (0)	1 (2)		0 (0)	
Minus-plus	1 (9)	18 (39)		12 (32)	
**Onda T em V4. n (%)**					0,005
Positiva	10 (91)	27 (59)		13 (35)	
Negativa	1 (9)	10 (22)		21 (57)	
Indeterminada	0 (0)	2 (4)		0 (0)	
Plus-minus	0 (0)	1 (2)		1 (3)	
Minus-plus	0 (0)	6 (13)		2 (5)	
**Onda T em V5. n (%)**					0,49
Positiva	10 (91)	34 (74)		22 (59)	
Negativa	1 (9)	8 (17)		12 (32)	
Indeterminada	0 (0)	1 (2)		1 (3)	
Plus-minus	0 (0)	2 (4)		2 (5)	
Minus-plus	0 (0)	1 (2)		0 (0)	
**T Orientation V6. n (%)**					0,62
Positiva	9 (82)	36 (78)		26 (70)	
Negativa	2 (18)	6 (13)		9 (24)	
Indeterminada	0 (0)	1 (2)		0 (0)	
Plus-minus	0 (0)	3 (6)		2 (5)	
Minus-plus	0 (0)	0 (0)		0 (0)	

Teste ANOVA

Ao se comparar os valores encontrados com os valores estimados extraídos do estudo de Davignon et al. ^2^ ( Tabela 4 ), detectamos diferenças estatisticamente significativas em vários parâmetros em todos os grupos etários (≤24 horas, entre 25e 72 horas, e entre 73 e 168 horas de vida), tais como amplitude das ondas P, Q, R e S, duração do QRS, e relação entre R e S (R/S e R+S).

**Tabela 4:** Razão de amplitude/duração de alguns parâmetros eletrocardiográficos entre o estudo de Pimenta et al. e o de Davignon et al.2

Variável	Horas de vida		

	<24	25-72	73-168
**Amplitude da onda P (DII)**	0,36	0,18	0,23
**Amplitude da onda Q**			
DIII	3,41	2,83	2,76
aVF	3,33	2,30	3,00
V5	1,10	1,4	1,10
V6	1,13	1,6	2,40
**Amplitude da onda R**			
V2	0,22	0,33	0,29
V5	0,41	0,18	0,8
V6	3,15	2,60	2,28
**Amplitude da onda S**			
V2	0,33	0,36	0,36
V4	0,25	0,40	0,37
V6	1,68	2,68	1,48
**Duração do complexo QRS (V5)**	2,53	0,07	0,26
**R / S**			
V1	0,91	0,86	0,85
V5	0,73	0,85	0,83
V6	0,82	0,90	0,89
**R + S (V2)**	0,29	0,36	0,34
**S em V1 + R (V6)**	1,87	1,64	1,39
**Intervalo PR (DII)**	0,11	0,6	
**Amplitude da onda R**			
aVR		1,32	
V1		0,19	
V5	1,41	1,18	
**Amplitude da onda S (V5)**		0,21	0,31
**Ondas R + S (V4)**		0,21	0,24
**Ondas S em V2 + R (V5)**		0,67	0,17

## Discussão

Diferentemente da população adulta, estudos eletrocardiográficos no período neonatal são escassos. Em 1979, Davignon et al. ^2^ publicaram achados de ECG de 2141 crianças, 549 com idade inferior a sete dias. Até hoje, esse é o maior estudo em recém-nascidos, e a maioria das diretrizes de interpretação de ECG em neonatos são baseadas nesse estudo. No entanto, não há provas de que os neonatos estudados de fato não tinham malformações cardíacas que pudessem influenciar parâmetros de ECG.

É esperado que alterações eletrocardiográficas ocorram nos primeiros dias de vida devido a importantes mudanças circulatórias nesse período. Assim, Davignon et al. ^2^ dividiram os recém-nascidos em três grupos (≤24 horas, entre 25 e 72 horas, e entre 73 e 168 horas de vida). Em nosso estudo, foram observadas diferenças significativas na direção da onda T nas derivações V1, V2, V3 e V4 entre os mesmos grupos etários. A maior proporção de ondas T positivas nos grupos mais novos pode ser explicada pela maior pressão pulmonar nessa fase, levando a uma repolarização inicial do VD. Com a diminuição fisiológica na pressão pulmonar que ocorre nos primeiros de vida, pode-se esperar uma mudança na repolarização para o padrão infantil, levando a uma proporção menor de ondas T nas derivações precordiais (V1 a V4). A análise da onda T não foi realizada no trabalho de Davignon. Não houve diferença estatística nos outros parâmetros eletrocardiográficos estudados.

Na comparação dos nossos resultados com os valores obtidos do estudo de Davignon et al., ^2^ observamos diferenças estatisticamente significativas em vários parâmetros em todos os grupos etários, em particular nas amplitudes de ondas (P, Q, R,S), duração do QRS e relação entre R e S (R/S e R+S). Calculamos uma razão simples em alguns parâmetros de ECG entre nossos resultados e os de Davignon, para enfatizar as diferenças encontradas (como mencionamos acima) – Tabela 4 .

Essas diferenças indicam que os parâmetros de normalidade de ECG propostos no estudo de Davignon et al. ^2^ podem não ser os mais adequados para a interpretação de ECG de recém-nascidos brasileiros hoje. ^11^ Além da possível diferença antropométrica entre populações (Canadá x Brasil), no estudo canadense, ^2^ não houve rastreamento de doença cardíaca, exames de imagem ou acompanhamento dos recém-nascidos. Portanto, não há evidências de que, de fato, a população do estudo de Davignon et al. ^2^ não apresentava nenhuma doença cardíaca estrutural.

Os resultados obtidos no presente estudo puseram em questão a aplicabilidade dos parâmetros eletrocardiográficos de normalidade relatados por Davignon et al. ^2^ para recém-nascidos a termo de até sete dias de vida, para outras nacionalidades e etnias.

### Limitações

A realização de um ECG em um recém-nascido é limitada por uma série de dificuldades, como o pequeno tamanho do tórax para posicionamento dos eletrodos, e o fato de serem extremamente agitados. Assim, decidimos que todos os exames seriam executados pelo mesmo médico para minimizar a influência do posicionamento dos eletrodos. Isso levou a um número limitado de recém-nascidos estudados. É importante ressaltar que provavelmente mais diferenças sejam encontradas se um maior número de recém-nascidos for estudado.

## Conclusão

Este é o primeiro estudo correlacionando achados eletrocardiográficos com ecocardiograma normal em uma coorte de recém-nascidos. Recém-nascidos a termo com até 24 horas de vida apresentaram significativamente mais ondas T positivas em comparação àqueles com mais horas de vida. Muitas diferenças foram encontradas na comparação com os parâmetros propostos por Davignon et al., ^2^ indicando que mais estudos são necessários para uma interpretação definita de ECG nos recém-nascidos.
