# Supplementary material for: Avaliação Eletrocardiográfica de Recém-Nascidos Normais na Primeira Semana de Vida – Estudo Observacional
Source: Arq Bras Cardiol. 2022 Aug 24;119(4):587–92. [Article in Portuguese] doi: 10.36660/abc.20210843 (PMC9563892; doi:10.36660/abc.20210843)
Supplement: Translation

# Translation: Electrocardiographic Evaluation of Normal Newborns in the First Week of Life – Observational Study

**Authors:** Marina de Souza Pimenta, Nelson Samesima, Carlos Alberto Pastore, Vera Lucia Jornada Krebs, Gabriela Nunes Leal, Werther Brunow de Carvalho

**Affiliations:** 1 Brazil Instituto da Criança (ICr) – Hospital das Clínicas da Faculdade de Medicina, Universidade de São Paulo, São Paulo, SP – Brazil; 2 Brazil Instituto do Coração (InCor) – Hospital das Clínicas da Faculdade de Medicina, Universidade de São Paulo, São Paulo, SP – Brazil

**Keywords:** Electrocardiography, Myocytes, Cardiac, Infant, Newborn

## Abstract

**Background:**

The neonatal period is marked by major changes in the cardiovascular system, especially in the first week of life. Unlike the adult population, studies on electrocardiogram (ECG) data in the neonatal period are scarce. This is the first study to describe electrocardiographic changes in a cohort of newborns with normal echocardiograms.

**Objectives:**

To analyze the electrocardiographic patterns of a population of full-term NB, without any cardiac morphological or functional anomalies, and compare the results with the literature.

**Methods:**

In this observational study, echocardiograms and ECG results from 94 newborns divided in three age groups (up to 24 hours, between 25 and 72 hours, and between 73 and 168 hours of life) were evaluated and compared with those reported by Davignon et al. A p-value <0.05 was considered statistically significant.

**Results:**

There were significant differences in T-wave direction in leads V1 (p= 0.04), V2 (p= 0.02), V3 (p= 0.008) and V4 (p= 0.005) between the three age groups. There were differences between our findings and the current literature in most of the parameters.

**Conclusion:**

Term newborns within 24 hours of life showed significantly more positive T waves than older ones. Many differences from the Davignon’s ECG parameters were found, particularly in the P, Q, R, S amplitudes, QRS duration, R/S and R+S. These findings indicate that more studies are needed for a definitive interpretation of the ECG in newborns.

## Introduction

The neonatal period is marked by many hemodynamic and anatomical cardiovascular changes, especially in the first week of life, when the transition of the circulation pattern from fetal to neonatal occurs.^1 , 2^ In the fetus, the placenta is a low-resistance vascular bed and the right ventricle (RV) is the dominant ventricle, responsible for approximately 60% of the cardiac output. The heart works with an almost constant workload, with a high-volume and low-resistance circulation. After the cut of the umbilical cord and the first breath there is a decrease in the pulmonary vascular resistance and an increase in the systemic vascular resistance. Right ventricular pressure and flow drops while the afterload increases as the placenta is removed and the left ventricular (LV) outflow increases by two-fold due to the increased pulmonary blood flow. The *foramen ovale* and *ductus arteriosus* close and the ventricular predominance change from the RV to the LV, with subsequent increase in cardiomyocyte size and number.^1 , 3^

It is not known whether these circulatory changes in the first days of life can lead to different patterns in the electrocardiogram (ECG). Hemodynamic changes are assessed in the clinical practice through clinical parameters (heart rate, oxygen saturation, respiratory pattern, heart auscultation) and complementary exams (e.g. echocardiogram, serum lactate and sodium bicarbonate). However, unlike the adult population, electrocardiographic studies in the neonatal period are scarce.^4^

The objective of this study was to describe the ECG findings in term neonates without cardiac malformations and normal cardiovascular function during hospital stay, and to compare them with the findings by Davignon et al.^2^ This is the first study correlating ECG findings with normal echocardiogram in a cohort of newborns.

## Methods

### Population

In this observational study, from August 2016 to July 2018, ECG results and echocardiograms of newborns during their first seven days of life (168 hours) were evaluated, all born at a tertiary neonatal unit in São Paulo, Brazil.

### Ethics

The study was approved by the Institutional Review Board (approval number 272/13/2016; CAPPesq 1.662.356) and conducted in accordance with the Declaration of Helsinki. The Ethics Commission waived the need for a patient’s consent form since the ECG and echocardiographic examinations are routine at the neonatal unit.

The inclusion criteria were gestational age (GA) between 37 and 41 weeks and 6 days, and less than 169 hours of postnatal age. Cardiac malformation was excluded using echocardiogram in the first 169 hours of life. All newborns had normal cardiovascular function during hospital stay.

Newborns with major non-cardiac malformations, such as neurological and chromosomal abnormalities, GA less than 37 weeks or equal to or above 42 weeks, or with abnormalities in the echocardiogram such as cardiac malformations (complex heart anomaly, valve dysfunction, major sept defect, aortic coarctation), persistent pulmonary hypertension, functional impairment or with abnormal cardiovascular function during hospitalization were excluded.

Newborns were divided by post-natal age in three groups: up to 24 hours, between 25 and 72 hours and between 73 and 168 hours of life to enable the comparison of our findings with those reported by Davignon et al.^2^

### 12-Lead ECG

Simultaneous twelve-lead ECG (Philips PageWriter TC20©, Koninklijke Philips, N.V.) was performed and analyzed in all neonates by a single trained not blinded investigator. Solid gel tab electrodes were positioned on the right and left shoulders, right and left iliac crests and V1-V6 as recommended by guidelines^5^ ( Figure 1 ). The shoulders and iliac crests were preferred over the right/left arms and legs due to the commonly excessive movement of the newborn, in order to reduce noise and improve ECG signals.^6^

**Figure 1:** Positioning of electrocardiogram electrodes in newborns

The following parameters were assessed:

- heart rate (bpm, automatically measured by the device),
- frontal plane QRS axis (°),
- P wave amplitude (mm) and duration (ms) and duration of PR interval in DII (ms),
- amplitude of: Q wave in DIII, aVF and V5-V6, R wave in aVR, V1-V2 and V4-V6, and S waves in V1-V2 and V4-V6 (mm),
- R/S ratio in V1 and V6,
- QRS duration, QT and QTc interval (corrected by Bazzet’s formula) in V2 (ms), and
- T wave duration (ms) and orientation (+ / -) in all 12 leads.

### Echocardiogram

A detailed two-dimensional echocardiography with Doppler was performed in all subjects by the on-call experienced pediatric cardiologist. The equipment used was a Philips CX50 (Koninklijke Philips N.V.), with multifrequency transducers S8-3 and S12-4. M-mode echocardiographic measurements of left atrium, RV, left ventricle, posterior wall, and LV diastolic and systolic diameters were obtained following the American Society of Echocardiography guidelines.^7^ LV ejection fraction was obtained by Teichholz method and was considered normal when equal to or greater than 55%.

### Statistical analysis

Qualitative characteristics of mothers and newborns were described as absolute and relative frequencies. Quantitative characteristics were described using summary measures (mean and standard deviation) for all subjects.^8 , 9^ ECG parameters were described according to maternal diseases such as hypertension (none, primary, gestational, primary + gestational) and diabetes mellitus (none, type 1, type 2, gestational). Based on their birth weight, the newborns were classified as small for GA (SGA), appropriate for GA (AGA), or large for GA (LGA); the values were described as summary measures and compared for the categories of interest using analysis of variance (one-way ANOVA) followed by Bonferroni multiple comparisons when p<0.05.^10^

The Kolmogorov-Smirnov data distribution normality test was performed, and the assumption of normality was accepted for most of the parameters evaluated. As this is a weaker assumption of ANOVA, it was conducted for all variables without loss of power in the analyses, since the central limit theorem guarantees the normality of distribution of the mean, even with no normality of the data.

The parameters were described in percentile curves and compared with normal values according to the percentiles reported by Davignon et al.^2^ The analyses were performed using the IBM-SPSS for Windows version 22.0 software. The value of p<0.05 was considered significant.

## Results

During the study period, there were 2,883 live births in the neonatal unit. Of these, 1,916 were full-term newborns. Echocardiograms were performed in 753 babies; 310 of them were full-term newborns, of whom 191 did not have any significant anatomical changes.

ECGs of 113 newborns were performed, 19 of whom were excluded because of major non-cardiac malformations, mainly anomalies of the central nervous system or genetic syndromes. The final series of the present study consisted of 94 patients.

Clinical characteristics of the newborns are presented in Table 1 . The percentiles of the ECG parameters studied are in Table 2 . In the comparison between-age groups (see Table 3 ), newborns with less than 24 hours of life had a significantly higher proportion of positive T waves compared to NB in the older groups (25–72 hours and 73–168 hours) in leads V1 (p = 0.04), V2 (p = 0.02), V3 (p = 0.008), and V4 (p = 0.005).

**Table 1:** Clinical characteristics of the newborns evaluated in the study (n=94)

Variables	Number (%)
**Age groups (hours of life)**	
≤24 hours	11 (12)
25-72 hours	46 (49)
73-168 hours	37 (39)
**Classification (body weight)**	
SGA	9 (10)
AGA	77 (82)
LGA	8 (8)
**Delivery**	
Vaginal	31 (33)
Forceps	9 (10)
Cesarean	54 (57)
**Gender**	
Female	53 (56)
Male	40 (43)
Indeterminate	1 (1)
	average (SD)
GA (weeks)	38.6 (1.1)
**Weight at birth (grams)**	3,184 (551)

GA: gestational age; SGA: small for GA; AGA: appropriate for GA; LGA: large for GA; SD: standard deviation.

**Table 2:** Percentiles of the electrocardiographic parameters

Parameter	<24 hours of life			24-72 hours of life			73-168 hours of life		

	5%	50%	95%	5%	50%	95%	5%	50%	95%
Heart Rate (bpm)	92.14	122.09	152.04	98.91	122.72	146.53	102.18	131.05	159.92
Ampl P DII (mm)	0.04	0.11	0.18	0.06	0.13	0.21	0.06	0.13	0.21
PR DII (ms)	70.55	92.73	114.91	71.43	99.13	126.83	73.40	98.38	123.36
QT V2 (ms)	227.59	301.82	376.04	206.84	293.48	380.12	202.20	274.05	345.90
QRS axis ( ° )	54.61	126.36	198.12	61.59	128.75	195.91	58.09	134.44	210.79
Ampl Q DIII (mm)	0.03	0.41	0.79	0.05	0.34	0.64	0.02	0.36	0.70
Ampl Q aVF (mm)	0.00	0.30	0.65	0.00	0.23	0.47	0.00	0.27	0.59
Ampl Q V5 (mm)	0.00	0.10	0.28	0.00	0.04	0.15	0.00	0.10	0.29
Ampl Q V6 (mm)	0.00	0.13	0.32	0.00	0.06	0.18	0.00	0.12	0.31
Ampl R aVR (mm)	0.00	0.33	0.83	0.00	0.33	0.71	0.00	0.25	0.66
Ampl R V1 (mm)	0.15	1.25	2.35	0.42	1.13	1.85	0.38	1.13	1.88
Ampl R V2 (mm)	0.56	1.35	2.14	0.46	1.19	1.92	0.43	1.27	2.11
Ampl R V4 (mm)	0.76	1.74	2.71	0.76	1.57	2.38	0.78	1.57	2.37
Ampl R V5 (mm)	0.35	1.41	2.48	0.51	1.30	2.09	0.49	1.29	2.09
Ampl R V6 (mm)	0.39	1.26	2.12	0.36	1.17	1.98	0.43	1.14	1.85
Ampl S V1 (mm)	0.24	1.00	1.75	0.00	0.97	2.10	0.00	0.67	1.37
Ampl S V2 (mm)	0.57	1.40	2.23	0.15	1.34	2.52	0.12	1.08	2.04
Ampl S V4 (mm)	0.00	1.21	3.23	0.07	0.97	1.87	0.08	0.88	1.67
Ampl S V5 (mm)	0.00	0.77	2.10	0.00	0.75	1.59	0.09	0.62	1.15
Ampl S V6 (mm)	0.00	0.59	1.66	0.00	0.67	1.51	0.08	0.52	0.96
R/S V1	0.24	1.37	2.50	0.00	2.10	5.87	0.00	2.38	5.46
R/S V5	0.00	5.26	18.79	0.00	3.03	8.63	0.00	3.41	11.42
R/S V6	0.00	5.31	17.78	0.00	3.32	9.46	0.00	3.17	8.20
R + S V2 (mm)	-9.16	-0.46	8.25	-11.73	-1.44	8.86	-7.37	1.88	11.13
R + S V4 (mm)	-14.88	5.23	25.33	-3.11	5.98	15.07	-3.29	6.97	17.24
S V2 + R V5 (mm)	1.68	2.81	3.94	1.19	2.64	4.09	1.14	2.38	3.61
S V1 + R V6 (mm)	1.01	2.25	3.50	0.72	2.14	3.55	0.88	1.81	2.73
Dur QRS V5 (ms)	40.00	40.00	40.00	30.60	44.78	58.97	27.41	46.49	65.56

Ampl: amplitude; bpm: beats per minute; Dur: duration; hl: hours of life; mm: millimeters; ms: milliseconds.

**Table 3:** Electrocardiographic T-wave parameters by age groups

Variable	Hours of life				p
	≤24	25-72	73-168		
T-wave in V1. n (%)					0.04
Positive	5 (45)	8 (17)		2 (5)	
Negative	3 (27)	23 (50)		23 (62)	
Minus-plus	3 (27)	15 (33)		12 (32)	
T-wave in V2. n (%)					0.02
Positive	6 (54)	7 (15)		4 (11)	
Negative	4 (36)	21 (46)		23 (62)	
Minus-plus	1 (9)	18 (39)		10 (27)	
**T-wave in V3. n (%)**					0.008
Positive	8 (73)	12 (26)		5 (13)	
Negative	2 (18)	15 (33)		20 (54)	
Plus-minus	0 (0)	1 (2)		0 (0)	
Minus-plus	1 (9)	18 (39)		12 (32)	
**T-wave in V4. n (%)**					0.005
Positive	10 (91)	27 (59)		13 (35)	
Negative	1 (9)	10 (22)		21 (57)	
Indeterminate	0 (0)	2 (4)		0 (0)	
Plus-minus	0 (0)	1 (2)		1 (3)	
Minus-plus	0 (0)	6 (13)		2 (5)	
**T-wave in V5. n (%)**					0.49
Positive	10 (91)	34 (74)		22 (59)	
Negative	1 (9)	8 (17)		12 (32)	
Indeterminate	0 (0)	1 (2)		1 (3)	
Plus-minus	0 (0)	2 (4)		2 (5)	
Minus-plus	0 (0)	1 (2)		0 (0)	
**T-wave in V6. n (%)**					0.62
Positive	9 (82)	36 (78)		26 (70)	
Negative	2 (18)	6 (13)		9 (24)	
Indeterminate	0 (0)	1 (2)		0 (0)	
Plus-minus	0 (0)	3 (6)		2 (5)	
Minus-plus	0 (0)	0 (0)		0 (0)	

ANOVA test

When comparing the values found with the estimated values extracted from the study by Davignon et al.^2^ ( Table 4 ), we noticed statistically significant differences in several parameters in all age groups (<24 hours, between 25 and 72 hours and between 73 and 168 hours of life), such as amplitude of P, Q, R and S waves, QRS duration and R-S relationship (R/S and R+S).

**Table 4:** Electrocardiographic standards ratio between Pimenta et al. and Davignon et al. 2 for newborn

Variable	Hours of life		

	<24	25-72	73-168
**P wave amplitude (DII)**	0.36	0.18	0.23
**Q wave amplitude**			
DIII	3.41	2.83	2.76
aVF	3.33	2.30	3.00
V5	1.10	1.4	1.10
V6	1.13	1.6	2.40
**R wave amplitude**			
V2	0.22	0.33	0.29
V5	0.41	0.18	0.8
V6	3.15	2.60	2.28
**S wave amplitude**			
V2	0.33	0.36	0.36
V4	0.25	0.40	0.37
V6	1.68	2.68	1.48
**QRS complex duration (V5)**	2.53	0.07	0.26
**R / S**			
V1	0.91	0.86	0.85
V5	0.73	0.85	0.83
V6	0.82	0.90	0.89
**R + S (V2)**	0.29	0.36	0.34
**S in V1 + R (V6)**	1.87	1.64	1.39
**PR interval (DII)**	0.11	0.6	
**R wave amplitude**			
aVR		1.32	
V1		0.19	
V5	1.41	1.18	
**S wave amplitude (V5)**		0.21	0.31
**R + S waves (V4)**		0.21	0.24
**S waves in V2 + R (V5)**		0.67	0.17

## Discussion

Unlike the adult population, electrocardiographic studies in the neonatal period are scarce. In 1979, Davignon et al.^2^ published ECG findings of 2,141 children, 549 of them under seven days of life. So far, this is the largest study on newborns, and most guidelines of ECG interpretation in neonates are based on this study. Nevertheless, there is no proof that the newborn studied, in fact, had no cardiac malformation that could influence ECG parameters.

It is expected that ECG changes occur in the first days of life, due to significant circulatory changes in this period. Thus, Davignon et al.^2^ divided the newborns in three age groups (<24 hours, between 25 and 72 hours and between 73 and 168 hours of life). In our study, significant differences were found in the direction of the T waves in leads V1, V2, V3 and V4 between the same age groups. The higher proportion of positive T waves in the younger age groups can be explained by the higher pulmonary pressure found in this early phase, leading to an initial repolarization of the RV. With the physiological drop in pulmonary pressure that occurs in the first days of life, a change in ventricular repolarization to the infantile pattern can be expected, leading to a lower proportion of positive T waves in precordial leads (V1 to V4). T wave analysis was not done in Davignon’s work. There was no statistical difference in the other electrocardiographic parameters studied.

When comparing our results with the estimated values extracted from the study by Davignon et al.,^2^ we observed statistically significant differences in several parameters in all age groups, particularly in wave amplitudes (P, Q, R, S), QRS duration and R-S relationship (R/S and R+S). We did a simple ratio within some ECG parameters between our results and Davignon’s to emphasize the differences found (mentioned above) – Table 4 .

These differences indicate that the parameters of electrocardiographic normality proposed by Davignon et al.^2^ may not be the optimal ones for interpreting ECG of Brazilian newborns today.^11^ In addition to the possible anthropometric difference between populations (Canada x Brazil), in the Canadian study,^2^ there were was no cardiac screening, image examination or follow-up of the newborns. Therefore, no evidence was presented that, in fact, the study population in their study did not have any structural heart disease.

The results obtained in the present study have called into question the applicability of the electrocardiographic parameters of normality reported by Davignon et al.^2^ for term newborns with up to seven days of life, to other nationalities and ethnicities.

### Limitations

The performance of an ECG on a newborn is fraught with difficulties, since besides dealing with their small chest size to deploy the electrodes, they are also highly agitated. In this way, we decided to have all the ECGs performed by the same physician, to minimize the influence of electrodes positioning. This led to a limited number of newborns studied. It is important to note that it is likely that more differences will be found if a greater number of newborns are studied.

## Conclusion

This is the first study correlating ECG findings with normal echocardiogram in a cohort of newborns. Term newborns within 24 hours of life showed significantly more positive T waves than the elders. Many differences from the parameters proposed by Davignon et al.^2^ were found and indicate that more studies are needed for a definitive interpretation of the ECG in newborns.
